# 

**Published:** 1871-11

**Authors:** 


### TABLE OF CONTENTS.

### ORIGINAL COMMUNICATIONS.

Paracentesis Thoracis—Recovery. By A. N. Talley, M.D., Professor of the Practice of Medicine in the University of South Carolina, Columbia. . . . . . . . . . . . . . 429

Uterine Cloth Tents. By V. H. Taliaferro, M.D., of Columbus, Georgia. . . . . . . . . . . . . . . . . . . . . . . . . . . . . 431

Meningitis Cerebro-Spinalis Epidemica. Translated by Ch. Raushenberg, M.D., Atlanta, Georgia . . . . . . . . . . 444

Case of Severe Wound of the Brain—Loss of Two Fluid-Ounces of Brain Substance, a Large Amount of Bony Covering, and Recovery without Impairment of Mind. By Wm. Abram Love, M.D., Atlanta, Georgia . 451

External Application of Chloral Hydrate. By Chas. S. Strother, M.D., Barnesville, Georgia. . . . . . . . . . . . . . 458

Reviews. By Wm. Abram Love, M.D., Atlanta, Georgia 460

Atlanta Academy of Medicine. J. T. Johnson, M.D., Reporter . . . . . . . . . . . . . . . . . . . . . . . . . . . . . . . . . . . . . . 463

### SELECTED ARTICLES.

On Hydrophobhia. (The American Practitioner). . . . . . . 466

Meddlesome Midwifery. By Francis H. Milligan, M.D., Wabash, Minnesota. (Northwestern Medical and Surgical Journal) . . . . . . . . . . . . . . . . . . . . . . . . . . . . . . . . . . 473

Belladonna in Orchitis. . . . . . . . . . . . . . . . . . . . . . . . . . . . 475

### EDITORIAL AND MISCELLANEOUS.

To Contributors . . . . . . . . . . . . . . . . . . . . . . . . . . . . . . . . . . . 476

Editorial Correspondence. . . . . . . . . . . . . . . . . . . . . . . . . . 477

Publications Received. . . . . . . . . . . . . . . . . . . . . . . . . . . . . 481

Rush Medical College. . . . . . . . . . . . . . . . . . . . . . . . . . . . . . 485

Selections. . . . . . . . . . . . . . . . . . . . . . . . . . . . . . . . . . . . . . . . 488

